# Transcriptome and metabolome analyses reveal molecular insights into waterlogging tolerance in Barley

**DOI:** 10.1186/s12870-024-05091-8

**Published:** 2024-05-09

**Authors:** Feifei Wang, Zhenxiang Zhou, Xiaohui Liu, Liang Zhu, Baojian Guo, Chao Lv, Juan Zhu, Zhong-Hua Chen, Rugen Xu

**Affiliations:** 1 Key Laboratory of Plant Functional Genomics of the Ministry of Education/Jiangsu Key Laboratory of Crop Genomics and Molecular Breeding/Jiangsu Co-Innovation Center for Modern Production Technology of Grain Crops/Institutes of Agricultural Science, Yangzhou University, Yangzhou, 225009 China; 2 College of Food and Pharmaceutical Engineering, Guizhou Institute of Technology, Guiyang, 550003 China; 3 School of Science, Hawkesbury Institute for the Environment, Western Sydney University, Penrith, NSW 2751 Australia

**Keywords:** *Hordeum vulgare* L., Hypoxia, Waterlogging stress, Metabolites, Multi-omics, Phenylpropanoid biosynthesis, Ethanol fermentation

## Abstract

Waterlogging stress is one of the major abiotic stresses affecting the productivity and quality of many crops worldwide. However, the mechanisms of waterlogging tolerance are still elusive in barley. In this study, we identify key differentially expressed genes (DEGs) and differential metabolites (DM) that mediate distinct waterlogging tolerance strategies in leaf and root of two barley varieties with contrasting waterlogging tolerance under different waterlogging treatments. Transcriptome profiling revealed that the response of roots was more distinct than that of leaves in both varieties, in which the number of downregulated genes in roots was 7.41-fold higher than that in leaves of waterlogging sensitive variety after 72 h of waterlogging stress. We also found the number of waterlogging stress-induced upregulated DEGs in the waterlogging tolerant variety was higher than that of the waterlogging sensitive variety in both leaves and roots in 1 h and 72 h treatment. This suggested the waterlogging tolerant variety may respond more quickly to waterlogging stress. Meanwhile, phenylpropanoid biosynthesis pathway was identified to play critical roles in waterlogging tolerant variety by improving cell wall biogenesis and peroxidase activity through DEGs such as *Peroxidase* (*PERs*) and *Cinnamoyl-CoA reductases* (*CCRs*) to improve resistance to waterlogging. Based on metabolomic and transcriptomic analysis, we found the waterlogging tolerant variety can better alleviate the energy deficiency via higher sugar content, reduced lactate accumulation, and improved ethanol fermentation activity compared to the waterlogging sensitive variety. In summary, our results provide waterlogging tolerance strategies in barley to guide the development of elite genetic resources towards waterlogging-tolerant crop varieties.

**Supplementary Information:**

The online version contains supplementary material available at 10.1186/s12870-024-05091-8.

## Background

Global climate change has led to more frequent flooding and waterlogging events, causing the reduction of productivity of arable farmland, substantial economic losses, and food security issues [1, 2]. As increasing number of global farming regions become more exposed to flooding, it is urgent to investigate the biology of flooding resilience in plants. A better understanding on how plants respond to waterlogging with differential expression of core stress-related genes and metabolic adjustments is critical to selecting and designing waterlogging resilient crop varieties.

Soil waterlogging causes prolonged periods of hypoxia/anoxia through the slow diffusion of oxygen in water and competition of the roots with respiring microorganisms, thus severely affects plant growth and productivity [3]. Hypoxia hampers aerobic respiration and ATP synthesis to limit the availability of required energy to fuel the H^+^-ATPase pumps, severely hindering the plant’s ability to deliver water and nutrients from roots to the shoots [4, 5]. To survive in low-oxygen environments, plants develop many morphological and anatomical alterations, such as the formation of aerenchyma [6, 7], a barrier against radial oxygen loss [1, 8] and the development of more adventitious roots [9, 10]. Moreover, plants have also evolved various strategies including changes in physiology and metabolism, such as improved anaerobic respiration, phytohormone-induced resistance and intense metabolic activities to cope with waterlogging stress [11–14].

When O_2_ levels decline in plants, hypoxic cells subsequently rely on glycolysis for energy maintenance [15]. To maintain the glycolytic flux, fermentation pathways are initiated to regenerate nicotinamide adenine dinucleotide (NAD^+^), which quickly depletes the plant’s carbohydrates and contributes to cytosolic acidification [16]. However, hypoxia tolerant plants can activate alanine (Ala) synthesis to limit this significant carbon loss [13, 17] and initiate the γ-aminobutyric acid (GABA) shunt that assists in the stabilization of the cytosolic pH [18]. During these carbohydrate metabolism processes, enzymes, including alcohol dehydrogenase (ADH), pyruvate decarboxylase (PDC), lactate dehydrogenase (LDH), and Ala aminotransferase (AlaAT) were reported to play important roles in plant responses to hypoxia and waterlogging stress [19, 20].

The Hordeum species including barley (*Hordeum Vulgare*) are widespread in temperate, subtropical, and subarctic areas, from sea level to heights of more than 4,500 m in the Andes and Himalayas, demonstrating high degrees of adaptation to different adverse environments [21]. This suggests that the barley and wild barley gene pool contains genetic diversity for environmental adaptability and stress resistance [22–25]. However, barley is more susceptible to waterlogging stress than other cereals, mainly showing decreased plant biomass, chlorophyll content and grain yield [26–28]. The screen for waterlogging tolerance varieties in barley has revealed 48 quantitative trait loci (QTL), which mainly located at linkage groups 2H, 3H and 4H [29]. One major QTL for aerenchyma formation was found to explain 44.0% of the phenotypic variance which can be effectively used in the marker assisted selection to improve waterlogging tolerance in barley [30, 31]. Moreover, one QTL for ROS tolerance in barley was identified on chromosome 2H which explained 23% and 24% of the phenotypic variation for O_2_^•−^ and H_2_O_2_ contents, respectively [32].

The responses of plants to environmental stresses are complex processes, and the rapid advancement of multi-omics technologies allows the in-depth investigation on how plants coordinate these intricate processes across multiple omics levels to achieve abiotic stress tolerance [33–35]. However, the mechanism of barley resistance to waterlogging has not been comprehensively investigated. We hypothesize that waterlogging tolerant barley variety show more differentially expressed genes (DEGs) and differential metabolites (DM) in transcriptional and metabolic level under waterlogging stress. We propose that the tolerant variety regulate metabolic reactions to improve energy availability to adapt to the low oxygen stress. To test the hypothesis, we conducted transcriptome and metabolome analyses in the leaf and root of two barley varieties showing contrasting waterlogging tolerance in our previous studies [32, 36–38]. The main objective of this study is to explore molecular adjustments in transcripts and metabolites in response to waterlogging stress in roots and shoots of barley varieties with contrasting waterlogging tolerance.

## Methods

### Plant growth conditions and waterlogging treatments

Two barley varieties NasoNijo (NN) and TX9425 (TX) were grown in the same pot (20 cm × 30 cm) in greenhouse with a 16 h (h)/8 h day/night regime in Yangzhou University, China. For each barley variety, six seeds were sown in each pot and totally three pots were used for control and another nine pots were used for three different waterlogging treatments. The seedlings were grown to three-leaf stage and then submerged in tap water for 1 h, 72 h and 2 weeks (w) according to Wang et al. [39]. After the waterlogging treatments, leaf and root samples from each of seedlings (5 to 6 seedlings used for one biological replicate with three biological replicates for one treatment) were collected for RNA-sequencing. After 2 w of waterlogging stress, the above-ground biomass of individual plants was measured as shoot fresh weight. Roots were gently washed and weighted. Leaf chlorophyll content was measured on the last third leaf of each seedling with a SPAD meter (SPAD-502, MINOLTA, Japan).

### RNA-sequencing analysis

The total RNA of NN and TX varieties in leaf and root was extracted after 1 h, 72 h and 2 w of waterlogging stress treatment with the RNeasy Plant Mini Kit (QIAGEN, Germany) and cDNA libraries were constructed using NEBNext Ultra RNA Library Prep Kit (NEB, USA). The libraries were detected by Qsep100 and sequenced by Illumina HiSeq TM2000 (San Diego, CA, USA). After removing the adapter sequences and low-quality reads, the clean reads were mapped to reference barley genome (Hordeum vulgare Morex V3, 2021) using HISAT2 (http://ccb.jhu.edu/software/hisat2). The average expression level of two biological replicates was calculated and the gene expression values were represented by log_2_(Fragments Per Kilobase of exon model per Million mapped fragments). The genes with |log_2_(Fold Change)|> 1.5 and *p*-value < 0.01 were regarded as DEGs. Kyoto encyclopedia of genes and genomes (KEGG) analysis [39–42] and gene ontology (GO) annotation of DEGs were conducted to identify the enrichment pathways and gene functions. Data were visualized with Venn plot and heatmap by TBtools software [43]. Four genes were randomly selected to perform qRT-PCR to validate RNA-sequencing data and the primer sequences were listed in Table S1. Total RNA was extracted from leaf and root of NN and TX varieties after 1 h, 72 h and 2 w of waterlogging stress treatment and RNA was reverse transcribed with a PrimeScript™ RT reagent Kit with gDNA Eraser Kit (TaKaRa, Dalian, China) and real-time PCR was performed with a TB Green Premix Ex Taq II Kit (TaKaRa, Dalian, China) using a CFX96 thermocycler (Bio-Rad, USA). The PCR program had two steps: one cycle of 95°C, 30 s; 40 cycles of 95°C, 5 s; 60°C, 30 s. Three biological and two technical replicates were performed for each treatment. *HvUPL* (*Ubiquitin-protein ligase*) was chosen as the reference gene, which was proved to be one of the suitable reference genes in barley under different abiotic stresses (osmotic, salt, heat, waterlogging) and hormonal treatments [26, 44].

### Metabolome analysis

NN and TX seeds were grown in the same condition as for RNA-sequencing experiment as each pot grown six seeds and six pots were used for three biological replicates. The leaf and root were collected after 2 w of waterlogging treatments for metabolome analysis. Samples of 200 mg fresh tissue was used for metabolites extraction and 20 µL from each sample were detected for quality control. The metabolites of samples were determined by chromatographic separation in an Thermo Ultimate 3000 system equipped with an ACQUITY UPLC® HSS T3 (150 × 2.1 mm, 1.8 µm, Waters) column and using a Thermo Q Exactive Plus mass spectrometer, according to De Vos et al. [45], Sangsteret al. [46] and Want et al. [47].

The raw data were converted in mzXML format with Proteowizard software (v3.0.8789) and XCMS tool of R (v3.3.2) was used for peak identification, extraction, and alignment, subsequently obtaining the data matrix with mass to charge ratio (m/z), retention time and peak intensity. In order to compare data of different magnitudes, batch normalization of peak intensity was performed. The metabolites with fold change of metabolite concentration more than 1.5-fold (*p* < 0.05) and VIP > 1 were regarded as differential metabolites compared to the control. The cluster analysis of differential metabolites and the enzyme genes expression which were significantly and differentially expressed in metabolite pathway were performed with TBtools.

### Statistic data analysis

**Fig. 1:**
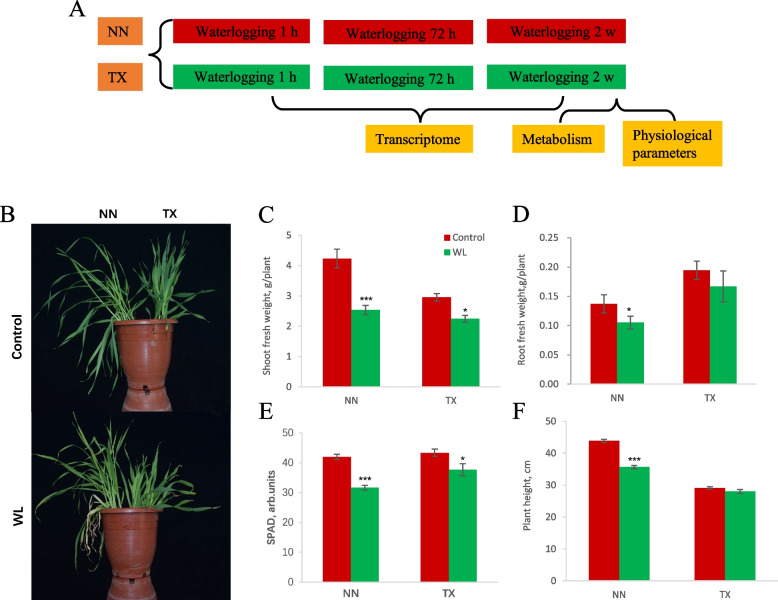
Scheme of the experiments performed (**A**). Three-leaf-stage seedlings of two varieties (NasoNijo, NN and TX9425, TX) were subjected to waterlogging stress for 1 h, 72 h and 2 weeks. The leaf and root were sampled after three treatments for RNA-sequencing analysis. After 2 weeks of waterlogging stress, physiological parameters were measured, and leaf and root were collected for metabolism analysis. Physiological responses to waterlogging stress in two barley varieties (**B**). Shoot (**C**) and root (**D**) fresh weight, leaf chlorophyll content (SPAD) (**E**) and plant height (**F**). Data are the mean ± SE (*n* = 12). The significance levels are *P < 0.05, **P < 0.01, and***P < 0.001

The physiological data in Fig. 1 are given as means ± SE. The significant differences were analyzed in Microsoft Excel software by paired samples t-test and the significance levels are *P < 0.05, **P < 0.01, and***P < 0.001. Principal component analysis (PCA) was performed to test main variable factors for contributing the total variation among samples in metabolite profiling using BioDeep website (https://www.biodeep.cn/tools/multianalysis?toolId=8).

## Results

### Distinct responses of two barley varieties to waterlogging stress

NasoNijo (NN) and TX9425 (TX) seedlings were grown to three-leaf stage and subjected to waterlogging stress for 1 h, 72 h and 2 w. The leaves and roots were sampled after three different treatments for RNA-sequencing analysis (Fig. 1A). Meanwhile, after 2 w of waterlogging stress, physiological parameters such as shoot and root fresh weight, leaf chlorophyll content and plant height were measured, and the leaves and roots were collected for metabolism analysis (Fig. 1A). The impact of waterlogging stress differed significantly between the NN and TX variety that the basal leaves of the NN seedlings were yellow and wilted, but only the leaf tips of TX appeared yellow (Fig. 1B). Shoot fresh weight (Fig. 1C) and leaf chlorophyll content (Fig. 1E) were significantly reduced in two barley varieties after 2 w of waterlogging stress, of which NN was more severely affected than TX. Specifically, the shoot fresh weight and chlorophyll content were decreased by 40.1% and 24.6% in NN; while in TX these reductions were only 23.9% and 13.0%, respectively (Fig. 1C, E). Moreover, there was no significant difference in root fresh weight and plant height after waterlogging stress in TX, whereas these two parameters in NN were significantly decreased by 23.4% and 18.7%, respectively, after treatment (Fig. 1D, F). Based on these results and previous publications [32, 36–38], the NN variety was more sensitive to waterlogging stress than TX.

### Waterlogging tolerant variety responds to waterlogging earlier with more DEGs in leaves

**Fig. 2:**
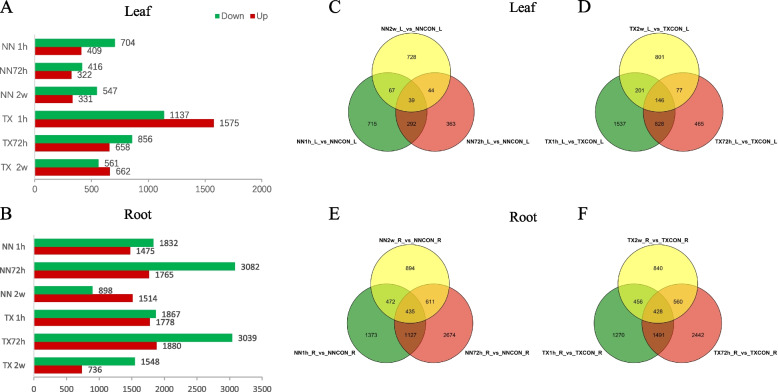
Numbers of up- and down- regulated differentially expressed genes (DEGs) in leaf (**A**) and root (**B**) of NN and TX variety after 1 h, 72 h and 2 w waterlogging stress. Fold change > 1.5-fold and *p* < 0.01 means the presence of significant difference in gene expression levels between two varieties. Venn plot of the overlap DEGs in leaf (**C**, **D**) and root (**E**, **F**) of two varieties after 1 h, 72 h and 2 w waterlogging stress, respectively

**Fig. 3:**
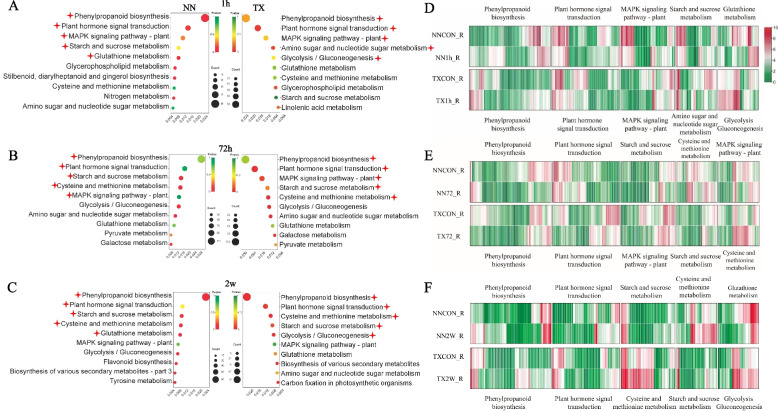
KEGG pathway enrichment [30–32] using KEGG database (https://www.kegg.jp/) of DEGs in root respond to 1 h (**A**), 72 h (**B**) and 2 w(**C**) waterlogging stress in NN and TX. Heatmap of DEGs in top 5 KEGG pathways after 1 h (**D**), 72 h (**E**) and 2 w(**F**) waterlogging stress in root of NN and TX

**Fig. 4:**
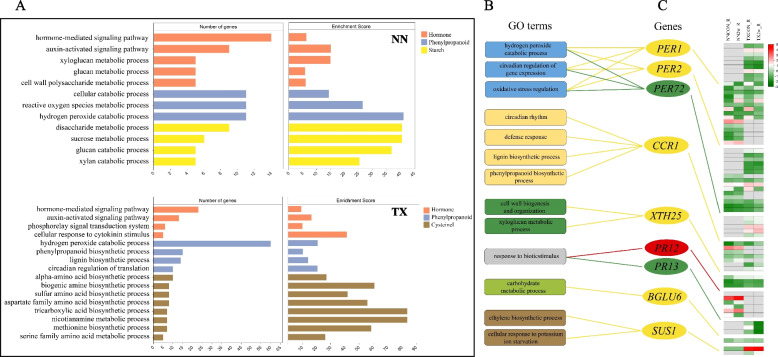
GO annotation of genes in top 3 pathways in NN and TX root after 2 w of waterlogging stress (**A**). GO terms from 3 pathways (**B**) and heatmap of related genes expression (**C**). *PER1*: peroxidase1, *PER2*: peroxidase2, *PER72*: peroxidase72, *CCR1*: cinnamoyl-CoA reductase 1, *XTH25*: xyloglucan endotransglucosylase/hydrolase protein 25, *PR12*: pathogenesis-related protein 12, *PR13*: pathogenesis-related protein 13, *BGLU6*: beta-glucosidase 6, *SUS1*: sucrose synthase 1. Note: The yellow line pointed to the gene (with yellow oval) means both NN and TX varieties had this gene; the green line pointed to the gene (with green oval) means only TX variety had this gene; the red line pointed to the gene (with red oval) means only NN variety had this gene

The transcriptome dataset was used to uncover intricate developmental and stress responses of two barley genotypes under waterlogging treatments (Figs. 2, 3 and 4). We performed RNA-sequencing of 32 samples of leaves and roots after 1 h, 72 h and 2 w of waterlogging treatments of the waterlogging sensitive (NN) and tolerant (TX) varieties, resulting in a total of 1.76 billion clean reads (255.95 Gb), and on average 96.66% of the clean reads were mapped to the *Hordeum vulgare* Morex V3, 2021 reference genome. Four DEGs (*PFK3, GST, RBOHB, MT11*) were randomly selected and their expression levels in leaf and root after 1 h, 72 h and 2 w of waterlogging treatment were quantified by quantitative real-time PCR (qRT-PCR). We found the qRT-PCR results were similar to those obtained in RNA-seq, indicating the reliability and reproducibility of the transcriptome dataset (Fig. S1).

The variation trend of DEGs was similar in the leaves of two varieties that there were largest numbers of up- and down-regulated DEGs at the early stage (1 h) of waterlogging stress compared to 72 h and 2 w treatment (Fig. 2A). Comparing different varieties, the numbers of up- and down-regulated DEGs in TX variety were higher than NN variety in leaves after three treatments (Fig. 2A). Especially, the numbers of up- and down-regulated DEGs in TX variety were 3.85 and 1.62-fold of NN variety after 1 h of waterlogging stress (Fig. 2A). In addition, there were 39 and 146 shared DEGs in leaves of NN and TX variety after three treatments, respectively (Fig. 2C, D). In roots, the DEGs numbers were substantially increased compared to the leaf in two varieties; particularly after 72 h of waterlogging stress that the increased folds of DEGs numbers ranged from 2.86 to 7.41 compared to leaf (Fig. 2A, B). Specifically, the down-regulated gene number in roots was 7.41-fold of that in the leaves of NN variety after 72 h of waterlogging stress (Fig. 2A, B). The number of DEGs in the two varieties were increased in the roots with the extension of waterlogging stress period to 72 h but decreased after 2 w of treatment (Fig. 2B). In both varieties, the number of downregulated genes exceeded that of upregulated genes after 1 h and 72 h of treatment in roots (Fig. 2B). Particularly, after 72 h of treatment, the down-regulated gene numbers were 1.62 to 1.75-fold of the up-regulated gene numbers in root of NN and TX, respectively (Fig. 2B). After 2 w of waterlogging stress, the number of down-regulated genes in the roots were still more than up regulated genes in TX, but NN showed the opposite trend (Fig. 2B). Intriguingly, the shared DEGs numbers among the three treatments in NN roots (435) were close to that in TX roots (428) (Fig. 2E, F). In summary, the numbers of up- and down-regulated DEGs in the roots were more than that in the leaves in both varieties, and the DEGs numbers in waterlogging tolerant TX variety were higher than that in NN variety in both leaf and root at the early stage of waterlogging stress (1 h).

### DEGs are highly enriched in the Phenylpropanoid biosynthesis pathway in the roots for waterlogging tolerance

Waterlogging stress dramatically remodeled the transcriptome in barley roots and leaves. KEGG analysis showed that DEGs were mainly enriched in “plant hormone signal transduction”, “MAPK signaling pathway”, “phenylpropanoid biosynthesis”, “starch and sucrose metabolism” and “glutathione metabolism” in leaf and root of both varieties (Fig. 3, Fig. S2). It was noteworthy that in the roots of both barley varieties the most enrichment pathway was phenylpropanoid biosynthesis under three different waterlogging treatments (Fig. 3A, B, C). In this pathway, around 18% of DEGs encoded peroxidases were down regulated after 1 h of treatment and this number increased to 50% when treatment was extended to 72 h in two varieties (Fig. 3D, E). GO annotation identified the top 3 pathways after 2 w of waterlogging stress, where we found hydrogen peroxide catabolic process which belonged to phenylpropanoid biosynthesis pathway enriched the highest number of DEGs (60) in the roots of TX (Fig. 4A). Moreover, the most of DEGs in hydrogen peroxide catabolic process were *Peroxidases 1* (*PER1*), *PER2* and *PER72*: the first two genes were found in both varieties and *PER72* was a DEG identified only in TX root (Fig. 4B, C). The numbers of up-regulated *PER1* and *PER2* genes in TX were more than those in NN (Fig. 4C). Moreover, the expression of *CCR1*(*Cinnamoyl-CoA reductase 1*) was increased after waterlogging stress in both varieties, and its expression in roots was higher in TX than in NN (Fig. 4C).

Another KEGG enrichment of DEGs in roots was starch and sucrose metabolism. *Sucrose synthase 1* (*SUS1*) was found to be up-regulated in the roots of both varieties and its expression was higher in TX than in NN after 2 w of waterlogging stress (Fig. 4C). Additionally, under the same treatment, large numbers of genes encoding aminotransferase such as *aspartate aminotransferase* (*ASP1*), *nicotianamine aminotransferase A, B* (*naat-A, B*) were found in cysteine and methionine metabolism pathway whose expressions were also higher in TX than in NN (Fig. 3F).

We also analyzed gene enrichment in leaves and the expression levels of genes such as *FERONIA (FER*) and *indole-3-acetic acid 6* (*IAA6)*, encoding receptor kinase and auxin related genes in the plant hormone signal transduction pathway were down regulated in NN and TX leaves after 1 h of waterlogging treatment (Fig. S2D). In glutathione metabolism, all *glutathione transferases* (*GSTs*) were down-regulated in NN leaves but in TX some *GSTs* were up-regulated after different waterlogging treatments (Fig. S2D, E, F). Many DEGs were enriched in the MAPK signaling pathway where *MAPKKK 17* (*Mitogen-activated protein kinase kinase kinase 17*) was down-regulated in both varieties after 2 w of waterlogging stress (Fig. S3C).

### Waterlogging stress generates disparate metabolites in roots and leaves

**Fig. 5:**
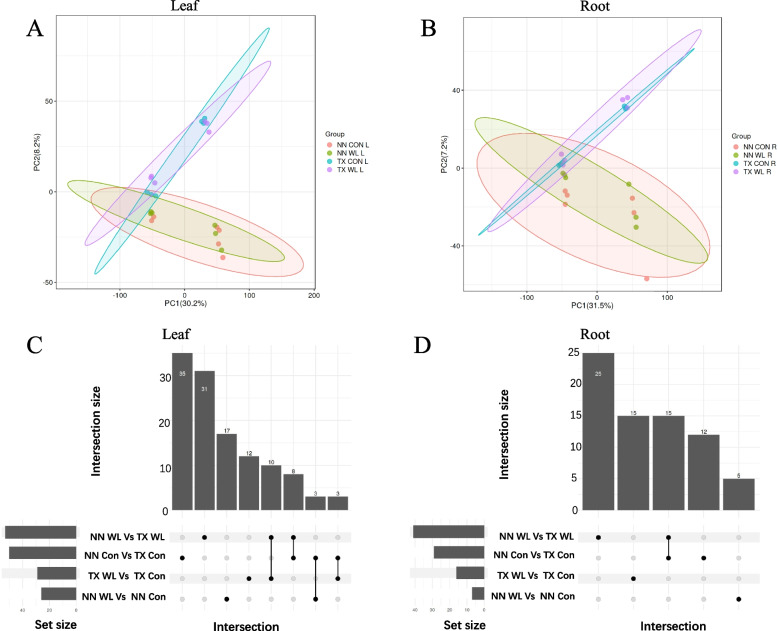
PCA analysis of all metabolome samples in leaf (**A**) and root (**B**) of NN and TX varieties under control and waterlogging stress. Upset Venn plots of differential metabolites in leaf (**C**) and root (**D**) of two barley varieties. The bar chart at the bottom-left represents the raw numbers of different groups; the dots and lines on the lower right represent the intersection between different groups; the bar chart at the top represents the number of intersections between different groups

The changes in genome, transcriptome, and proteome will eventually alter the metabolome, which is regarded as a mirror to phenotype. Therefore, we analyzed the metabolomic profiles in two barley varieties after 2 w of waterlogging stress using liquid chromatography-mass spectrometry (LC–MS). Principal component analysis (PCA) indicated that PC1 was not the major factor to separate two varieties in roots and leaves (Fig. 5A, B). In PC2 analysis, TX leaves and roots in stress conditions were separated from NN on either control or stress condition (Fig. 5A, B). Up-set Venn plot analysis showed that in leaf after waterlogging stress, NN and TX had 26 and 30 differential metabolites (DMs) compared to control (NN WL Vs NN Con, TX WL Vs TX Con; Fig. 5C), respectively. Meanwhile, there were 50 DMs between NN and TX in control condition (NN Con vs TX Con, 35 unique DMs; Fig. 5C) and 53 metabolites after waterlogging stress (NN WL vs TX WL, 31 unique DMs; Fig. 5C). Compared four groups in leaves, 8 metabolites including shikimic acid, GABA, pipecolic acid and 3,4-Dihydroxyphenylpropanoate were shared between NN WL vs TX WL and NN Con vs TX Con group (Fig. 5C). It was worth noting that the waterlogging stress induced numbers of DMs were less in roots than those in leaves in both varieties. Specifically, compared to control, there were only 16 and 7 DMs in roots of NN and TX; while there were 26 and 30 DMs in leaves of NN and TX, respectively (Fig. 5C, D). In roots, 28 and 41 DMs were found in control condition (NN Con vs TX Con; Fig. 5D) and waterlogging stress (NN WL vs TX WL, 25 of them were unique; Fig. 5D) as well as between NN and TX, respectively. Compared the four groups in roots, 15 DMs in roots was more than those in leaves identified between NN WL vs TX WL and NN Con vs TX Con group, including GABA, pipecolic acid, 3,4-Dihydroxyphenylpropanoate, tryptophan, cysteine (Fig. 5C, D).

**Fig. 6:**
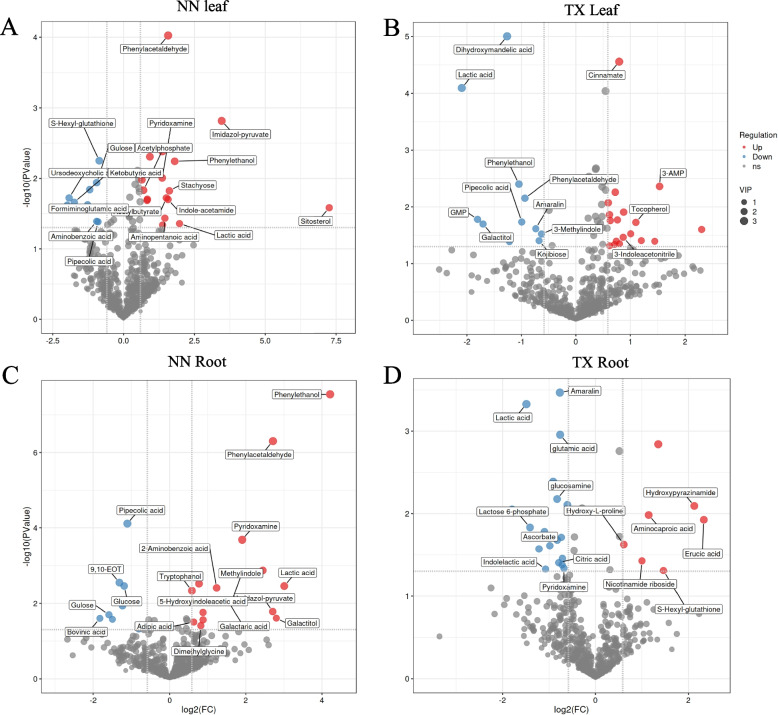
Volcanic plot of fold changes and VIP plot of metabolomic profiles in leaf (**A**, **B**) and root (**C**, **D**) of NN and TX, respectively. X axis represents the fold change of metabolite levels (WL / control). Y axis represents –log_10_ transformed *p*-value. Fold change > 1.5-fold, *p* < 0.05 and VIP > 1 indicate significant difference of metabolites. The red and blue dots represent up and down regulated metabolites in leaf and root of NN and TX, respectively. The size of dots represents the VIP size

Volcano and VIP score plots showed that the relative concentrations of metabolites, such as phenylpropanoid metabolites including phenylacetaldehyde, phenylethanol were significantly increased in leaves and roots of NN, but decreased or not changed in leaves and roots of TX, respectively (Fig. 6). Meanwhile, lactic acid was dramatically improved in NN but decreased in TX leaves and roots (Fig. 6). For sugars, we found, gulose concentration was all significantly declined in leaf and root; stachyose content was increased in leaf and glucose content was decreased in root of the NN variety (Fig. 6A, C). On the other hand, kojibiose was found to be significantly reduced in TX leaves (Fig. 6B). To better understand if changes in metabolites are distinct in roots and leaves upon oxygen deficiency, we used heatmap to show the concentration changes levels of metabolites, with 6 replicates for control and waterlogging treatment (Fig. S4). In the leaf of both varieties, most types of the DMs (18/27 in NN; 18/29 in TX) were significantly increased after waterlogging stress (Fig. S4A, B). In NN roots, most of the DMs were significantly increased, which included imidazol-5-yl-pyruvate, phenylacetaldehyde, lactic acid and phenylethanol; In contrast, in the TX variety, most of the DMs were significantly declined which included lactic acid, lactose 6-phosphate and ascorbate (Fig. S4C, D).

**Fig. 7:**
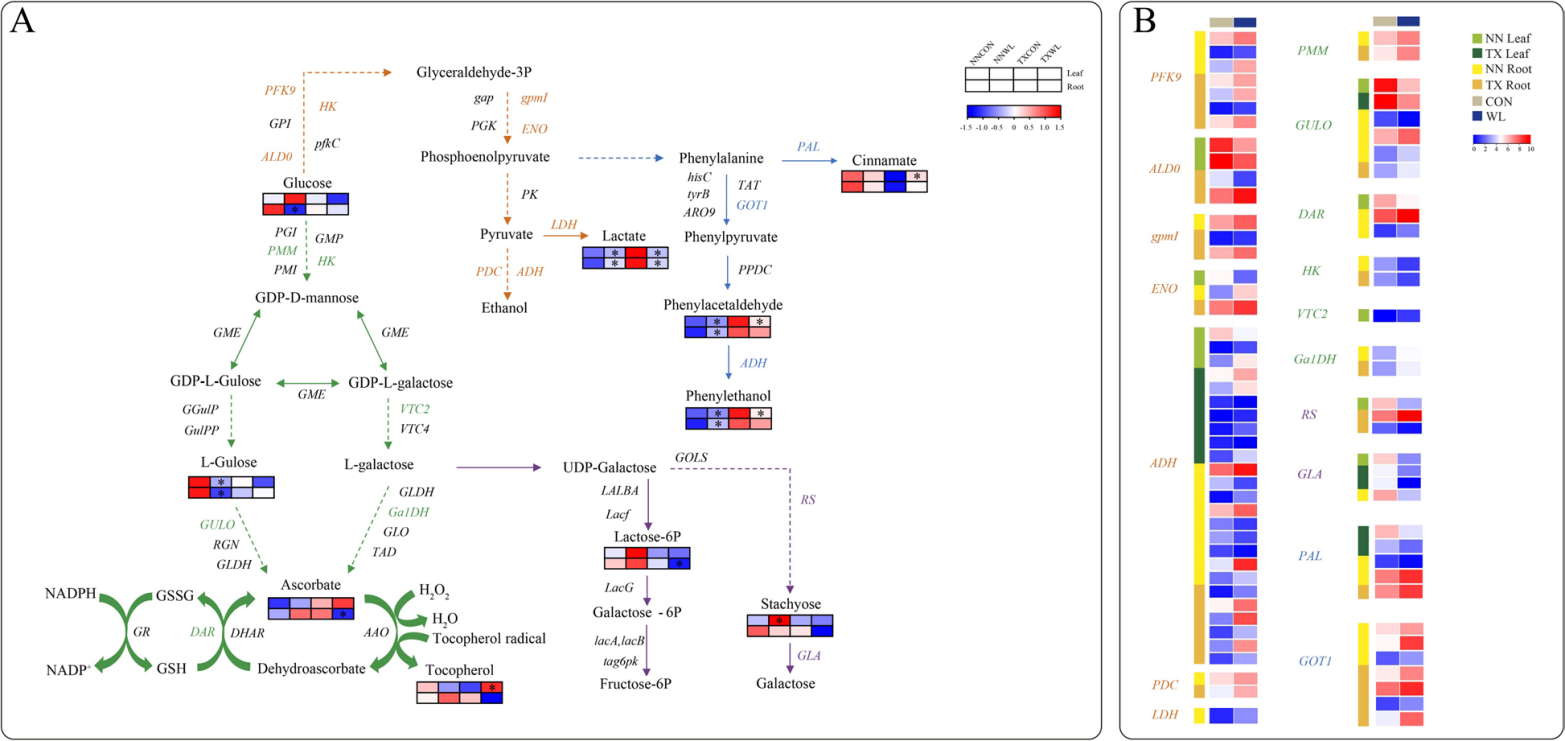
Accumulation of phenylalanine, lactate, galactose and ascorbate metabolic pathways with heat map of metabolite changes and relative enzyme genes expressions responding to waterlogging stress. The differential metabolite concentrations were performed in heatmap (**A**) as the first line of rectangles represents the changes in leaf of NN and TX under control and waterlogging stress while the second lines represent the changes in root. Enzymes with significantly different expressions in leaf and root of two barley varieties were marked in different color and corresponding expression levels were showed in heatmap (**B**). PFK9: 6-phosphofructokinase, ALD0: fructose-bisphosphate aldolase, pgmI: 2,3-bisphosphoglycerate-independent phosphoglycerate mutase, ENO: enolase, ADH: alcohol dehydrogenase, LDH: L-lactate dehydrogenase, PMM: phosphomannomutase, GULO: L-gulonolactone oxidase, DAR: dehydroascorbate reductase, HK: hexokinase, VTC2: GDP-L-galactose phosphorylase, GalDH: L-galactose dehydrogenase, RS: raffinose synthase, GLA: alpha-galactosidase, PAL: phenylalanine ammonia-lyase, GOT1: Glutamic-oxaloacetic transaminase 1. Asterisks indicate the significant differences at *P < 0.05, **P < 0.01, and***P < 0.001

One of the aims of this study was to decipher the difference in metabolism between waterlogging sensitive and tolerant varieties. We focused on the significantly DMs that are mainly involved in phenylalanine, lactate, galactose and ascorbate pathways, with heatmap to show the metabolites accumulation changes (Fig. 7A). Meanwhile, to identify relationships between metabolite and transcript regulation, we also measured the genes expressions under 2 w of waterlogging stress (Fig. 7B). In the ascorbate synthesis pathway, the glucose, gulose, ascorbate and tocopherol contents were found to be significantly changed after waterlogging stress (Fig. 7A). The accumulation of glucose and gulose were decreased by 56.06% and 66.70% in NN roots compared to the control, respectively, while the gulose concentration declined by 74.80% in NN leaves (Fig. 7A; Fig. S4A, C). In the waterlogging tolerant TX variety, the content of glucose and gulose were not significantly changed in either leaves or roots (Fig. 7A; Fig. S4B, D). However, the downstream metabolites such as ascorbate and tocopherol were found to be significantly different only in TX variety (Fig. 7A). Specifically, in TX roots, the ascorbate decreased by 49.40% and the tocopherol increased by 2.16-fold in leaf after waterlogging stress compared to the control (Fig. 7A; Fig. S4B). In this pathway, we found the expression of 6 key enzyme-encoding genes including *PMM* (*phosphomannomutase*), *GULO* (*L-gulonolactone oxidase*), *DAR* (*dehydroascorbate reductase*), *HK* (*hexokinase*), *VTC2* (*GDP-L-galactose phosphorylase*) and *GalDH* (*L-galactose dehydrogenase*) were significantly changed after waterlogging stress (Fig. 7B). For instance, the expression levels of *GULO* in leaves of both varieties were significantly declined after waterlogging stress whereas the transcripts of *GULO5* were decreased but the other two DEGs encoding *GULO2* were increased in NN roots (Fig. 7B). Interestingly, we found the expression levels of *HKs* were all declined in roots of both varieties (Fig. 7B).

In the lactate pathway, there were only two significantly different metabolites: glucose and lactate. The lactate response to waterlogging stress in barley showed variety specificity: the lactate contents in NN were increased by 2.92 and 7.05-fold, whereas these were decreased by 76.60% and 64.30% in the leaves and roots of TX, respectively (Fig. 7A). Moreover, the expression of *PFK9* (*6-phosphofructokinase*) in the lactate pathway was stimulated in the roots of both varieties under waterlogging stress (Fig. 7B). Meanwhile, the number of up-regulated *ADHs* in the leaf and root of TX variety was higher than those in NN, where the expression of *ADH3* was increased by 85.40-fold in the roots of TX (Fig. 7B). ADH enzyme also participated in the phenylalanine pathway. The contents of phenylacetaldehyde and phenylethanol were significantly increased in the leaves and roots of NN; whereas these two metabolites were significantly decreased in TX (Fig. 7A). We also found *GOT1* (*Glutamic-oxaloacetic transaminase 1*) response to waterlogging stress that the expression levels of *GOT1s* were all significantly increased in the roots of both varieties (Fig. 7B). The expression of *PAL1* (*Phenylalanine ammonia-lyase*) was decreased in the leaves of TX variety, but increased in the roots of both varieties, which was inconsistent with the relatively higher accumulation of cinnamate in leaf of TX (Fig. 7A, B). The accumulation of lactose-6-phosphate in the galactose pathway also showed variety specificity. In the leaves and roots of NN variety, the lactose-6-phosphate content increased while in the roots of TX the content was decreased by 62.34% (Fig. 7A).

## Discussion

### The waterlogging tolerant variety responds to waterlogging stress more quickly in the root

Not surprisingly, both varieties exhibited organ-specific differences in the leaves and roots, the number of DEGs was significantly higher in the root than in leaf. Particularly, after 72 h of waterlogging stress, the numbers of both upregulated and downregulated genes in the root increased by 2.86 to 7.41 times compared to the leaf. It may be possible that the leaves remain in the air, which have access to more oxygen, whereas the root is submerged in water with hypoxia stress that invokes more DEGs response to stress. Our result was similar to Arabidopsis under hypoxic stress [13]. Moreover, comparing the DEGs in waterlogging sensitive and tolerant varieties, the number of up- and down- regulated genes in the leaf of TX variety was higher than in NN variety after three treatments, particularly at the early stage of waterlogging stress (1 h). These results were similar to another study in barley that under 24 h of waterlogging stress, the number of both up- and down-regulated genes in the root was higher in waterlogging tolerant variety than in waterlogging sensitive variety, whereas the result reversed as the treatment time increased [48]. Interestingly, in wheat Shen et al. [49] also found the waterlogging tolerant variety has more DEGs than waterlogging sensitive variety. Taken together, we suggested the waterlogging tolerant barley variety may respond more quickly to waterlogging stress than the sensitive variety at the transcription level in order to gain advantages in the survival of hypoxia.

### Enhanced phenylpropanoid biosynthesis improves waterlogging tolerance ability

We used KEGG enrichment to gain an overview of the DEGs that were preferentially in function pathways. In our study, we found the amount of DEGs related to phenylpropanoid biosynthesis in both the leaves and roots of two barley varieties after waterlogging stress which was in accordance with the detection of the amount of phenylpropanoid biosynthesis pathway metabolites accumulation such as phenylacetaldehyde, phenylethanol and cinnamate. Especially in the roots, the numbers of DEGs in this pathway were the largest in two varieties after different waterlogging treatments compared to other pathways. Our results demonstrated the phenylpropanoid biosynthesis pathway plays important roles in response to waterlogging stress in barley. As one of the most important metabolism pathway in plants, phenylpropanoid metabolism yields more than 8,000 metabolites contributing to plant development and plant-environment interplay [50], such as heat, light, cold, drought as well as pathogen infection [51, 52]. Interestingly, the accumulation of DMs in phenylpropanoid biosynthesis showed strong variety-specific characters that under control condition the phenylacetaldehyde and phenylethanol concentrations were higher in the leaves and roots of TX variety than NN. It was also found in wild barley population, which was adapted to moist and fungi-rich soil and had higher phenylpropanoid/phenolamide biosynthesis abilities than the wild population which adapted to dry soil [34]. In general, based on the pivotal roles of phenylpropanoid metabolism in abiotic stress, we suggested that enhanced phenylpropanoid biosynthesis improves waterlogging tolerance ability in barley. However, waterlogging stress decreased these metabolites in TX variety but increased in NN. We proposed that the tolerant variety TX might further convert phenylacetaldehyde and phenylethanol into phenylacetate, which enters the downstream metabolic pathway to enhance TX’s tolerance to waterlogging.

Function annotation analysis showed that the numbers of up regulated *PER1, PER2* and *PER72* genes which reduce hydrogen peroxide and other hydroperoxides to water were higher in TX than in NN after waterlogging stress. Meanwhile, in this pathway we also found the expression of cell wall biogenesis and lignin biosynthesis related genes such as *CCR1*, which provides mechanical support to plant tissues and participates in the formation of vessels, subsequently enhancing waterlogging stress resistance [53, 54] were higher in TX than NN. In other barley varieties, Luan with his colleagues [48] also found the xyloglucan endotransglycosylase/hydrolase (XTH) enzymes genes which play a role in the loosening of cell walls and affect cell proliferation were significantly upregulated in waterlogging tolerant variety. This further proved that the waterlogging tolerance variety has higher cell wall biogenesis and peroxidase activity than waterlogging sensitive variety, which suggested the waterlogging tolerant plant resists waterlogging stress by its higher antioxidant activity and cell wall reinforcement ability.

### Waterlogging tolerant variety accumulate more sugars than sensitive variety under waterlogging stress

We found the DEGs number in “starch and sucrose metabolism” pathway was increased after 72 h of waterlogging stress in roots of both varieties. In this pathway, a large number of glycosyl hydrolase family genes such as *BGLU* (*Beta-glucosidase*) encoding glucosidase which catalyze the hydrolysis of starch or cellulose to produce low molecule sugars (e.g. glucose) displayed downregulation in both varieties after 72 h of waterlogging stress but upregulation with the extension of stress period. This was also found in Arabidopsis that after 2 h of hypoxia stress, 4.2% of mRNAs in the transcriptome and 63% of translatome was reduced; meanwhile, the root showed a greater reduction than shoot [55, 56]. When oxygen is limited in plants, starch hydrolyzes slowly under the action of amylase. However, under hypoxic or anoxic conditions, many plants must rely on hydrolyzed soluble sugar reserves for energy [56]. We found the expression of *BGLU6* and *SUS1* encoding sucrose synthases were all increased after 2 w of waterlogging stress in root of both varieties, which indicated that barley inhibited hydrolases catalyzation under short-term waterlogging stress but increased sugar production with the extension of stress period. Meanwhile, the expression of *BGLU6* was higher in NN than in TX root meanwhile the expression of *SUS1* was opposite suggesting two barley varieties adapted to waterlogging stress through different fine regulations.

Availability and mobility of sugar reserves are important for hypoxic organs. After 2 w of waterlogging stress, we found the accumulation of glucose and gulose compared to control were dramatically decreased in NN; whereas in TX they were not significantly changed in both leaf and root which suggested the waterlogging tolerant variety accumulated more sugars than the sensitive variety under waterlogging stress. In rice and wheat the glucose, fructose and sucrose contents were all decreased with anoxic stress; interestingly, the decrease rate of three kinds sugar in rice was lower than that of wheat [57] which was similar to our two barley varieties. In summary, it is indicated that barley inhibited hydrolases catalyzation under short-term waterlogging stress, but increased sugar production with the extension of stress period. The waterlogging tolerant variety accumulated more sugar than the sensitive variety, possibly owing to decelerating the rate of sugar consumption.

### Fortify ethanol fermentation activity to alleviate the energy deficiency in roots

When oxygen levels are insufficient to maintain the production of ATP via mitochondrial oxidative phosphorylation in plants, the rearrangement of metabolism involves increased catabolism of soluble carbohydrates for substrate-level production of ATP to maintain critical processes such as the activity of plasma and vacuolar membrane proton pumps that limit acidification of the cytosol [58, 59]. During these processes, plants elevate the mRNAs encoding enzymes that promote sucrose breakdown and entry into glycolysis, as well as the conversion of pyruvate to the fermentation end-products [55, 60]. Pyruvate fermentation produces energy in two different ways, producing lactic acid via LDH or producing ethanol via PDC and ADH [61]. Lactate and ethanol production are both disadvantageous to plant as lactate rapidly leads to cytosolic acidosis unless actively efflux of the cell and ethanol allows carbon to be lost by diffusion [62]. The lactate accumulation was measured in both varieties which showed variety specificity that in NN leaf and root the lactate contents were increased 3 to sevenfold; instead, the contents were decreased 64 to 77% in leaves and roots of TX variety. Interestingly, as the key enzyme in lactate pathway, the expression of LDH was up-regulated after waterlogging stress only in NN roots (Fig. 7B). It was reported that overexpression of LDH significantly enhanced the hypoxia resistance in Arabidopsis, whereas mutant *ldh* showed the opposite phenotype [63], suggesting that lactic acid fermentation was an important pathway in response to waterlogging stress. However, we found the waterlogging tolerant barley variety reduced the accumulation of lactate to improve the waterlogging tolerant ability. We did not measure ethanol in leaves and roots since it rapidly escaped to the atmosphere. The transcripts of *ADH* and *PDC* were more highly induced in roots of both barley varieties, which was also demonstrated in rice, wheat and Arabidopsis that roots produce more ethanol and have higher ADH and PDC activities than illuminated shoots under low-oxygen conditions [13, 57], suggesting greater ethanolic fermentation capacity in root. Meanwhile, the number and expression of up-regulated *PDC* and *ADH* genes of TX varieties were all higher than those of NN varieties. The higher expression of *PDC* genes in barley after waterlogging stress was also found previously, suggesting the waterlogging tolerant barley variety can accumulate more energy by decomposing more carbohydrates and amino acids [48]. Since PDC and ADH activities are usually considered as one of the important indexes reflecting the tolerance of plants to waterlogging [64], these results prove that waterlogging tolerant variety has higher ethanol fermentation activity to alleviate the energy deficiency in root than the sensitive variety.

## Conclusions

**Fig. 8:**
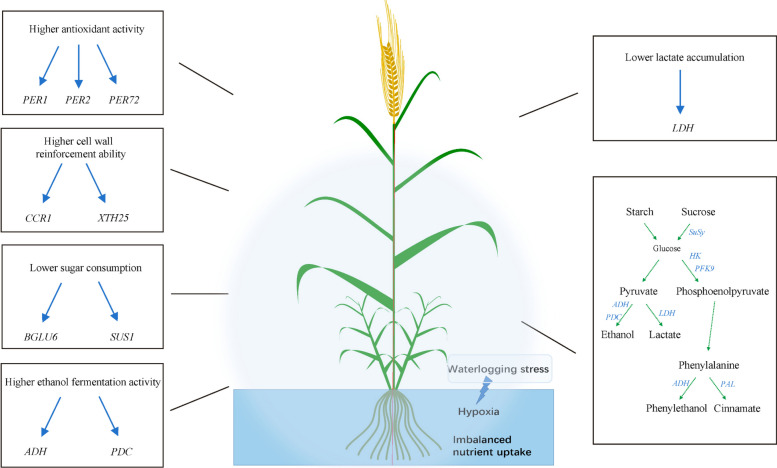
Schematic summary of differences in metabolism and related genes that were identified to waterlogging tolerance in barley

We propose a model of the various metabolism processes related to genes and metabolites in barley response to waterlogging stress (Fig. 8). First, barley showed strong organ-specific characteristics which were similar to other plants that the hypoxic-core response genes were more highly induced in roots than shoot under waterlogging stress; meanwhile, the tolerant variety responds more quickly and much stronger to waterlogging stress than sensitive variety at the transcription level. Second, the waterlogging tolerant variety was observed to have strong phenylpropanoid biosynthesis with higher cell wall biogenesis and peroxidase activity and a lower sugar consumption rate than sensitive variety. Plant survival under hypoxic conditions involves effective management of metabolic reconfiguration so that sugar reserves are not rapidly depleted under sustained stress. In barley we found the waterlogging tolerant variety reduced the accumulation of lactate to avoid cytosolic acidosis and improved ethanol fermentation activity to alleviate the energy deficiency. Our results provide new insights into physiological and molecular mechanisms underlying waterlogging stress in barley and genetic resources for barley breeding.

### Supplementary Information

Supplementary file 1.

Supplementary file 2.

Supplementary file 3.

## Data Availability

The transcriptome data presented in the study are deposited in the NCBI GEO repository, accession number GSE230751. The metabolism data presented in the study are deposited in the METABOLIGHTS repository, accession number MTBLS9380.

## Abbreviations

ADH: Alcohol dehydrogenase
AGT: Ala aminotransferase
ASP1: Aspartate aminotransferase
BGLU6: Beta-glucosidase 6
CAD5: Cinnamyl alcohol dehydrogenase 5
CCR1: Cinnamoyl-CoA reductase 1
CML46: Calmodulin-like 46
Control: Con
DAR: Dehydroascorbate reductase
DEGs: Differentially expressed genes
ENO: Enolase
GalDH: L-galactose dehydrogenase
GLA: Alpha-galactosidase
GOT1: Glutamic-oxaloacetic transaminase 1
GST: Glutathione S-transferase family protein
GULO: L-gulonolactone oxidase
HK: Hexokinase
KEGG: Kyoto encyclopedia of genes and genomes
LDH: L-lactate dehydrogenase
MAPKKK 17: Mitogen-activated protein kinase kinasekinase 17
MT11: Methyltransferase type 11
naat-A: Nicotianamine aminotransferase A
naat-B: Nicotianamine aminotransferase B
NN: NasoNijo
PAL: Phenylalanine ammonia-lyase
PCA: Principal component analysis
PDC: Pyruvate decarboxylase
PER1: Peroxidase1
PER2: Peroxidase2
PER72: Peroxidase72
PFK3: ATP-dependent 6-phosphofructokinase 3
PFK9: 6-Phosphofructokinase
pgmI: 2,3-Bisphosphoglycerate-independent phosphoglycerate mutase
PHT1: Putrescine hydroxycinnamoyl transferase 1
PMM: Phosphomannomutase
PR12: Pathogenesis-related protein 12
PR13: Pathogenesis-related protein 13
RBOHB: Respiratory burst oxidase homolog protein B-like
RS: Raffinose synthase
SPAD: Soil and plant analyzer development
SUS1: Sucrose synthase 1
SuSy: Sucrose synthase
TX: TX9425
VTC2: GDP-L-galactose phosphorylase
WL: Waterlogging
XTH25: Xyloglucan endotransglucosylase/hydrolase protein 25
