# Supplementary material for: Transcriptome and metabolome analyses reveal molecular insights into waterlogging tolerance in Barley
Source: BMC Plant Biol. 2024 May 9;24:385. doi: 10.1186/s12870-024-05091-8 (PMC11080113; doi:10.1186/s12870-024-05091-8)
Supplement: Supplementary file 1 [file 12870_2024_5091_MOESM1_ESM.docx]

**Supplementary Table 1.** Primer sequences for qRT-PCR.

| **Gene name** | **Primer sequence** |
| --- | --- |
| *PFK3-F* | 5’- CATTGATGCCGCTCATGTGG -3’ |
| *PFK3-R* | 5’- AGTCCACATCTCTGCTTGCC -3’ |
| *GST-F* | 5’- CGGTGGTGGACGAGAACGTA -3’ |
| *GST-R* | 5’- GTAGCGCATGAAGGGGAAGTG -3’ |
| *RBOHB-F* | 5’- CCACTCCAGAATCGCCCATC -3’ |
| *RBOHB-R* | 5’- AGGATATTTGCGTGCGCTTC -3’ |
| *MT11-F* | 5’- GGACAAGGGCATGGTGTCAT -3’ |
| *MT11-R* | 5’- TGTTCCAACACCAAGCTCCA -3’ |
| *UPL-F* | 5’- CTGAAGAGTTAGGCGGGAAA -3’ |
| *UPL-R* | 5’- ATCGCATGAACGTAGTGCAA -3’ |
