# Supplementary material for: Transcriptome and metabolome analyses reveal molecular insights into waterlogging tolerance in Barley
Source: BMC Plant Biol. 2024 May 9;24:385. doi: 10.1186/s12870-024-05091-8 (PMC11080113; doi:10.1186/s12870-024-05091-8)
Supplement: Supplementary file 3 [file 12870_2024_5091_MOESM3_ESM.pdf]

Supplementary figures:

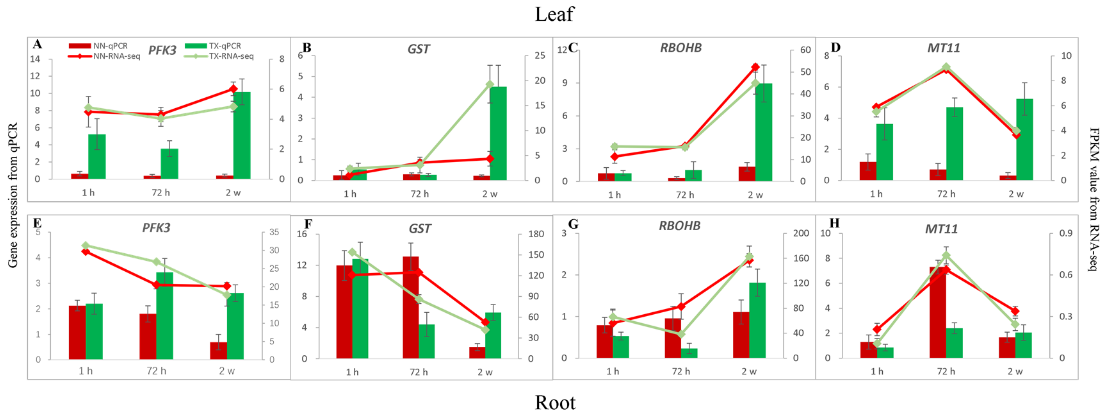

Fig. S1. qRT-PCR verification of the gene expressions of 4 randomly selected genes in RNA-seq data. *HvUPL* was used as reference gene in qRT-PCR analysis. Three biological replicates were applied for the leaf and root of each variety (mean ± SE).

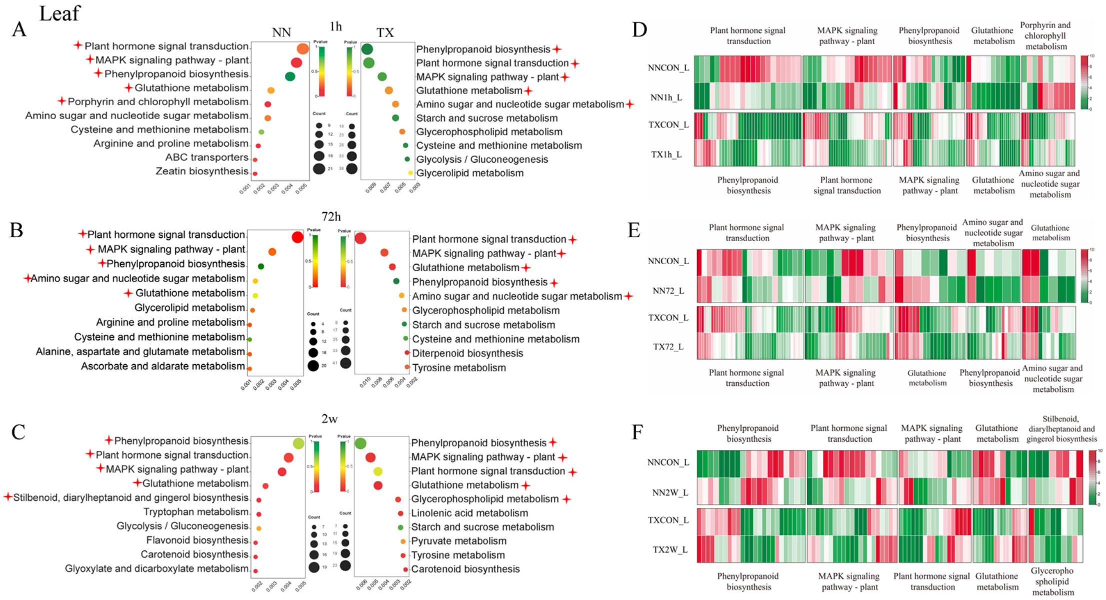

Fig. S2. KEGG pathway enrichment [30-32] using KEGG database (https://www.kegg.jp/) of DEGs in leaf respond to 1h (A), 72h (B) and 2 w (C) waterlogging stress in NN and TX. Heatmap of DEGs in top 5 KEGG pathways after 1h (D), 72h (E) and 2 w (F) waterlogging stress in leaf of NN and TX.

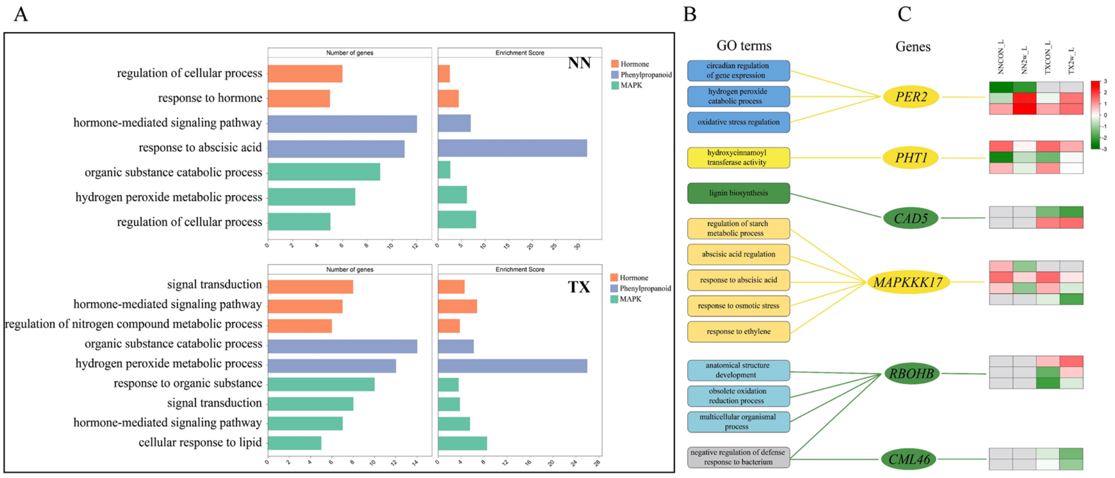

Fig. S3. GO annotation of genes in top 3 pathways in NN and TX leaf after 2 w of waterlogging stress (A). GO terms from 3 pathways (B) and heatmap of related genes expression (C). *PER2*: peroxidase 2, *PHT1*: putrescine hydroxycinnamoyl transferase 1, *CAD5*: cinnamyl alcohol dehydrogenase 5, *MAPKKK 17*: Mitogen-activated protein kinase kinase kinase 17, *RBOHB*: respiratory burst oxidase homolog protein B-like, *CML46*: calmodulin-like 46. Note: The yellow line pointed to the gene (with yellow oval) means both NN and TX varieties had this gene; the green line pointed to the gene (with green oval) means only TX variety had this gene; the red line pointed to the gene (with red oval) means only NN variety had this gene.

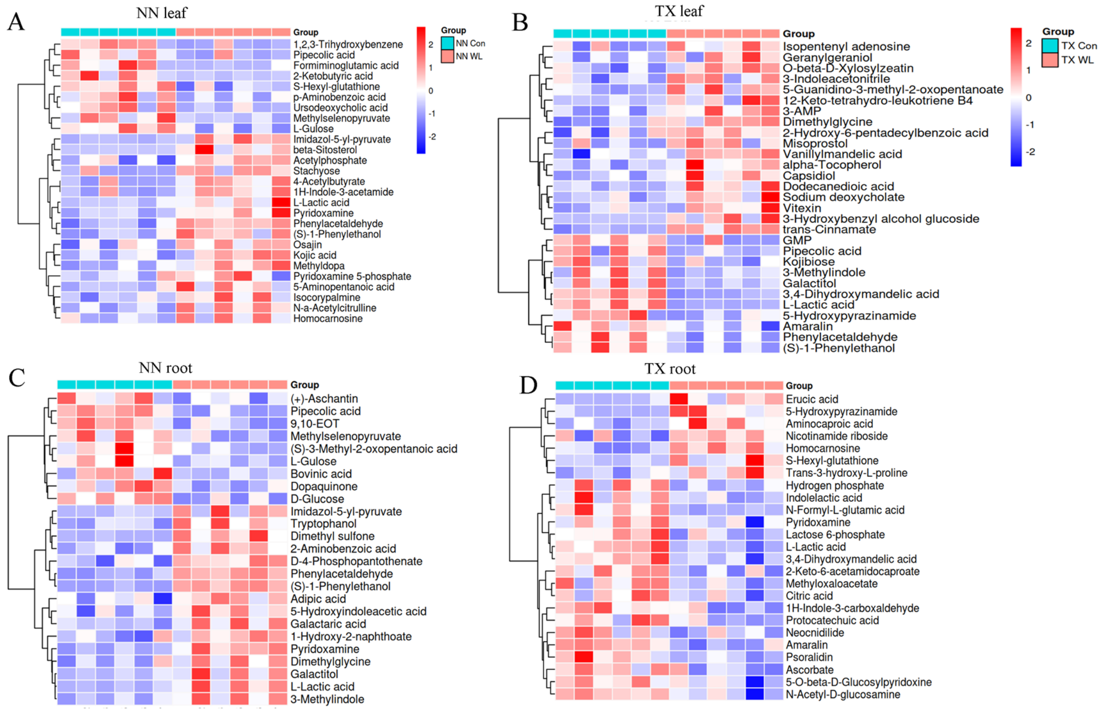

Fig. S4. Heatmap of significantly different metabolites in leaf (A, B) and root (C, D) of NN and TX variety after 2 w of waterlogging stress. Six replicates were measured for each treatment. The blue rectangle represents control (Con) condition, and the red rectangle represents waterlogging (WL) treatment.
